# Mimicry Embedding Facilitates Advanced Neural Network Training for Image-Based Pathogen Detection

**DOI:** 10.1128/mSphere.00836-20

**Published:** 2020-09-09

**Authors:** Artur Yakimovich, Moona Huttunen, Jerzy Samolej, Barbara Clough, Nagisa Yoshida, Serge Mostowy, Eva-Maria Frickel, Jason Mercer

**Affiliations:** a MRC-Laboratory for Molecular Cell Biology, University College London, London, United Kingdom; b Institute of Microbiology and Infection, University of Birmingham, Birmingham, United Kingdom; c Host-Toxoplasma Interaction Laboratory, The Francis Crick Institute, London, United Kingdom; d Department of Infection Biology, London School of Hygiene & Tropical Medicine, London, United Kingdom; e Section of Microbiology, MRC Centre for Molecular Bacteriology and Infection, Imperial College London, London, United Kingdom; University of Michigan-Ann Arbor

**Keywords:** capsule networks, transfer learning, superresolution microscopy, vaccinia virus, *Toxoplasma gondii*, zebrafish, deep learning

## Abstract

The use of deep neural networks (DNNs) for analysis of complex biomedical images shows great promise but is hampered by a lack of large verified data sets for rapid network evolution. Here, we present a novel strategy, termed “mimicry embedding,” for rapid application of neural network architecture-based analysis of pathogen imaging data sets. Embedding of a novel host-pathogen data set, such that it mimics a verified data set, enables efficient deep learning using high expressive capacity architectures and seamless architecture switching. We applied this strategy across various microbiological phenotypes, from superresolved viruses to *in vitro* and *in vivo* parasitic infections. We demonstrate that mimicry embedding enables efficient and accurate analysis of two- and three-dimensional microscopy data sets. The results suggest that transfer learning from pretrained network data may be a powerful general strategy for analysis of heterogeneous pathogen fluorescence imaging data sets.

**IMPORTANCE** In biology, the use of deep neural networks (DNNs) for analysis of pathogen infection is hampered by a lack of large verified data sets needed for rapid network evolution. Artificial neural networks detect handwritten digits with high precision thanks to large data sets, such as MNIST, that allow nearly unlimited training. Here, we developed a novel strategy we call mimicry embedding, which allows artificial intelligence (AI)-based analysis of variable pathogen-host data sets. We show that deep learning can be used to detect and classify single pathogens based on small differences.

## Abstract (precis)

In biology, the use of deep neural networks (DNNs) for analysis of pathogen infection is hampered by a lack of large verified data sets needed for rapid network evolution. Artificial neural networks detect handwritten digits with high precision thanks to large data sets, such as MNIST, that allow nearly unlimited training. Here, we developed a novel strategy we call mimicry embedding, which allows artificial intelligence (AI)-based analysis of variable pathogen-host data sets. We show that deep learning can be used to detect and classify single pathogens based on small differences.

## INTRODUCTION

Artificial neural networks (ANN) excel at a plethora of pattern recognition tasks, ranging from natural language processing (1) and facial recognition (2) to self-driving vehicles (3, 4). In biology, recent advances in machine learning and deep learning (5–7) are revolutionizing genome sequencing alignment (8), chemical synthesis (9, 10), and biomedical image analysis (11–14). In the field of computer vision, convolutional neural networks (CNNs) perform object detection and image classification at a level matching or surpassing that of human analysts (15).

Despite this, CNN-based architectures often poorly recognize unseen or transformed (e.g., rotated) data due to the use of maximum or average pooling (16). While pooling allows CNNs to generalize heterogenous data, positional information is ignored. This leads to prioritization of smaller image features and results in an inability of the network to “see the big picture.” To circumvent this, dynamically routed capsule-based architectures have been proposed (16, 17). These architectures are nested, allowing the retention of image feature positional information and optimization of CNN performance on images with a larger field of view.

However, these architectures remain data-hungry and often perform poorly on small host-pathogen data sets of high complexity (18). One major reason for this is the lack of large, balanced well-verified biological data sets (19), akin to MNIST (20) and ImageNet (21), that allow rapid algorithm evolution. To circumvent this, ANN analysis of pathogen images can be aided through transfer learning (22, 23). For this, weights of a network trained on one data set are transferred onto a fully or partially identical untrained network, which is then trained on a host-pathogen data set of a similar nature (23). This approach shortens training time and is generally considered more efficient than random weights initialization strategies (22).

Here, we describe a novel data embedding strategy, which we term “mimicry embedding,” that allows researchers to utilize ANNs in their analysis. Mimicry embedding involves transforming data sets such that they mimic verified nonbiomedical data sets, thereby allowing mimicry weights transfer from the latter. While most host-pathogen biomedical data sets are not large enough for deep learning, advances in high-content two-dimensional (2D) and 3D fluorescence imaging (24) can serve to increase the size of data sets for ANN analysis (14). By embedding 3D, fluorescent-image-based vaccinia virus and 2D/3D Toxoplasma gondii host-pathogen interaction data sets to mimic gray-scale handwritten digits, we demonstrate that mimicry weights transfer from MNIST (20) allows one to harness the performance of cutting-edge ANN architectures for the analysis of host-pathogen data sets.

## RESULTS

### Image acquisition and pathogen detection.

**FIG 1:**
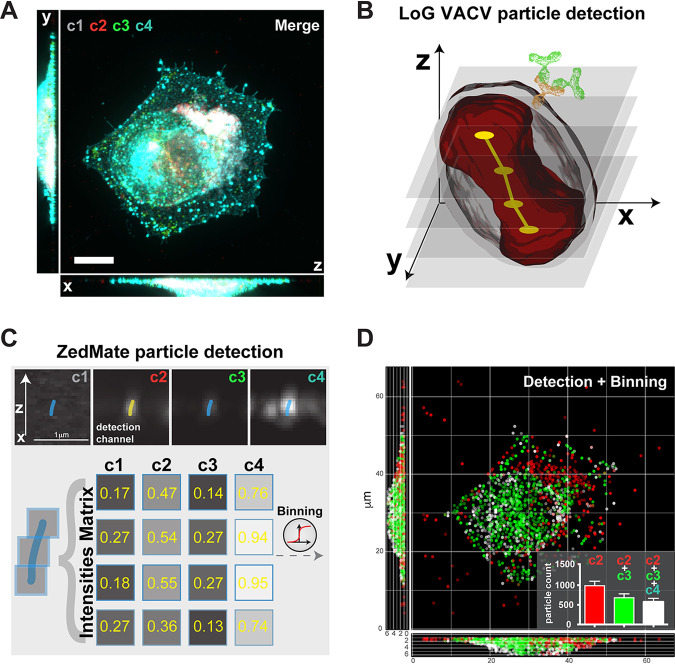
ZedMate facilitates detection and classification of VACV particles in infected cells. (A) Merged four-channel fluorescent image of a HeLa cell infected with VACV (see Fig. S1A for channel details). Bar, 10 μm. (B) Illustration of Laplacian of Gaussian (LoG)-based VACV particle detection in 3D. The dumbbell shape (red) represents a particle sliced in optical Z-sections (semitransparent gray), providing a point signal for LoG detection (yellow) and connected in Z (not to scale). (C) Intensity measurement from detected particles presented as a Z-profile intensity matrix. (D) 3D plot of detected particles color coded according to detected channels and virion category (see Fig. S1B for details). (Inset) Quantification of different particle types. *n* = 30 cells (3 biological replicates). Values are means and standard errors of the means (SEM).

To classify single-pathogen data in 3D biomedical images, we developed ZedMate, an ImageJ-Fiji (25) plugin that uses the Laplacian of Gaussian spot detection engine of TrackMate (26). We challenged ZedMate with multichannel, 3D fluorescent images of late-time-point vaccinia virus (VACV)-infected cells (Fig. 1A and Fig. S1). Owing to its large size, well-defined structure, and multiple layers of resident proteins that distinguish different virus forms, VACV has the features needed for complex fluorescence microscopy-based pathogen particle analysis (Fig. 1B). By detecting and linking individual virions within an image across the Z-dimension, ZedMate transforms a series of 2D images into a 3D data set (Fig. 1B). From the original four-fluorescent-channel composite, ZedMate generates grayscale images that preserve the intensity distribution across the Z-dimension of each detected channel (Fig. 1C, top). From this, fluorescence intensity matrices of each channel per Z-plane are then generated for individual particles (Fig. 1C, bottom). Using these matrices and accounting for the 3D positional information of the detected particles, ZedMate reconstructions can be plotted (Fig. 1D). Intensity analysis across all channels allows for binning (e.g., using a manual or automated threshold selection strategy) of virions into three categories consistent with their biological readouts (Fig. S1B).

10.1128/mSphere.00836-20.1 FIG S1 Individual channels used in late VACV-infected cells and their biological relevance. (A) Maximum-intensity projections of individual channel images of HeLa cells infected with VACV at 8 h postinfection. Channels are DNA stain (c1), VACV core A5-mCherry (c2), VACV outer envelope protein F13 (c3), and VACV outer envelope protein B5 (c4). Bar, 10 μm. (B) Illustration of the position of markers in virions (MV, mature virions; IEV, intracellular enveloped virions; CEV, cell-associated extracellular virions), and these virions, with the corresponding markers, in infected cells. Here, c1 marks cellular DNA and cytoplasmic VACV replication sites, c2 marks all virions (MV, IEV, and CEV), c3 marks a subset of virions (IEV and CEV), and c4 marks only CEV. Download FIG S1, TIF file, 2.1 MB. Copyright © 2020 Yakimovich et al. 2020 Yakimovich et al. This content is distributed under the terms of the Creative Commons Attribution 4.0 International license.

### Mimicry embedding assists deep neural network training for virus particle analysis.

**FIG 2:**
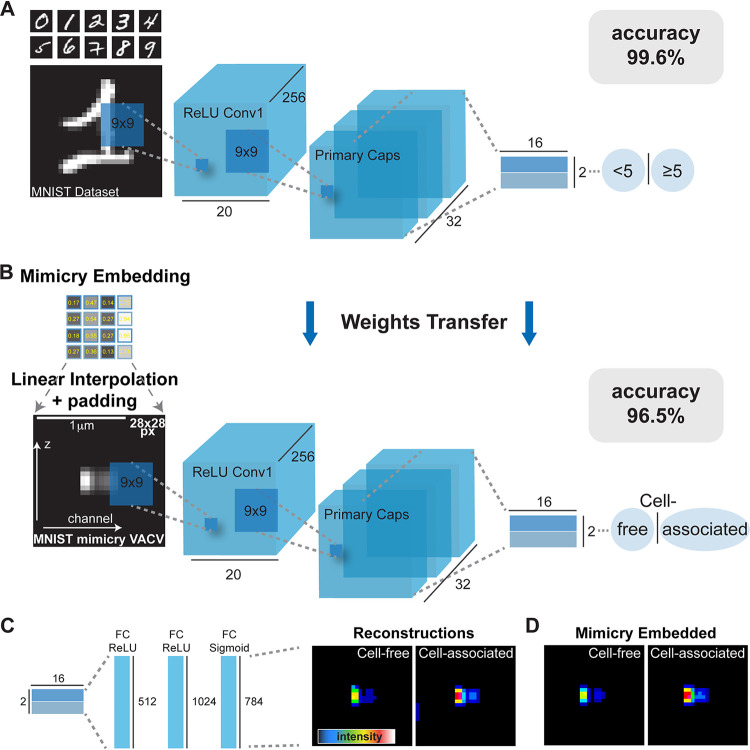
Mimicry embedding allows separation of cell-free and cell-associated VACV particles through weights transfer from a CapsNet trained on the binary MNIST data set. (A) CapsNet architecture for training on the MNIST handwritten digits data set repurposed into a binary classification problem (<5 or ≥5) prior to CapsNet weights transfer. Black numbers represent dimensions of tensors. ReLU, rectified linear unit. (B) Mimicry embedding of VACV Z-profiles detected by ZedMate. The intensity matrix of fluorescence signal (Fig. 1) was embedded to mimic MNIST data using linear interpolation and padding. Bar, 1 μm. CapsNet architecture with pretrained weights from A was used for training on mimicry-embedded VACV particles. (C) Reconstructed particle profiles of the virions separated into cell-free and cell-associated subsets by CapsNet. (D) Representative mimicry-embedded VACV particles for comparison to images in panel C. Statistical validation of machine learning models is provided in Fig. S3.

Initial reconstructions indicated that ZedMate cannot distinguish between incoming cell-free virions and newly replicated cell-associated virions based solely on channel 1 (c1) and c2 intensities (Fig. 1D and Fig. S1). To improve the precision of ZedMate-based binning, we devised a binary machine learning/deep learning (ML/DL) strategy relying on manual annotation to separate cell-free from cell-associated virions. Using random sampling, we generated a data set of 36,492 images containing equal numbers of cell-associated and cell-free virions. To maintain the spatial information acquired in ZedMate, we attempted to train the capsule ANN (CapsNet) (16) on this annotated data set. During initial attempts, CapsNet failed to converge, with accuracy around 0.5 (Fig. S2A). As CapsNet is known to converge well on the relatively simple grayscale data set, MNIST (16) (Fig. S2B), we asked how CapsNet would converge on a binary classification problem similar to ours. We tested this by splitting MNIST into balanced (<5 or ≥5; evens or odds) or unbalanced (one digit versus all) data sets. With no changes to CapsNet other than loading binary relabeled MNIST weights (<5 and ≥5) for initialization, CapsNet converged on MNIST with 99.6% accuracy (Fig. 2A). We found that all binary relabeled MNIST weights variants performed comparably (Fig. S2C).

10.1128/mSphere.00836-20.2 FIG S2 Relabeling the MNIST data set for weights transfer. (A) Accuracy of CapsNet trained on the late VACV data set with three different learning rates (LR) (0.1, 0.001, and 0.00001) fluctuates around 0.5 indicating no convergence. (B) Training loss, accuracy, and validation accuracy of CapsNet trained on the MNIST data set shows good and rapid convergence, while trained with similar parameters. (C) Training loss, accuracy, and validation accuracy of CapsNet trained on the MNIST data set relabeled for binary classification shows good convergence. Here, to test how binary classification can be performed on MNIST, we split the 10 digits into two halves (<5 and ≥5 [indicated as “half”] or odd and even numbers [indicated as “odd”]) or minor and major fractions (indicated as “unbalanced”). Download FIG S2, TIF file, 0.3 MB. Copyright © 2020 Yakimovich et al. 2020 Yakimovich et al. This content is distributed under the terms of the Creative Commons Attribution 4.0 International license.

10.1128/mSphere.00836-20.3 FIG S3 CapsNet training and validation of the cell-free versus cell-associated virus model. (A) Late model loss function change upon training iterations (epochs). (B) Late model training and validation (unseen data) accuracy change upon training iterations (epochs). (C) Late model receiver operational characteristics (ROC) curve of the trained model obtained using unseen data (validation). Here, the area under the curve (AUC) was 0.989. (D) Late model confusion matrix of the trained model obtained using unseen data (validation). Late model precision was 96.2%, recall was 96.2%, and the F1 score was 96.2%. Download FIG S3, TIF file, 0.2 MB. Copyright © 2020 Yakimovich et al. 2020 Yakimovich et al. This content is distributed under the terms of the Creative Commons Attribution 4.0 International license.

To allow transfer learning from this network to our VACV data set, we designed a vector-embedding strategy we term “mimicry embedding.” For this, the tensors of each virion’s multichannel, fluorescence Z-profiles from ZedMate are assembled across the *x* axis. This is followed by linear interpolation and padding, which serve to center the virion in a 28- by 28-pixel image such that the resulting data mimic the grayscale MNIST data set (Fig. 2B). With this approach, we aimed to preserve the weights of early CapsNet layers by maintaining the binary MNIST-trained CapsNet architecture and performing weights transfer. Training on our mimicry-embedded real-world data set achieved 96.5% accuracy (96.2% precision, 96.2% recall) at separating cell-free from cell-associated virions (Fig. 2B; see Fig. S3A to D for classifier training). Data set augmentation (10% horizontal and vertical shift), with mimicry embedding and MNIST weights transfer, resulted in only modest improvement to generalization, and there was no difference without mimicry embedding and MNIST weights transfer (data not shown).

To visualize how the trained ANN distinguished between cell-free and cell-associated virions with such accuracy, we used the CapsNet generator. The reconstructions indicated that cell-free virions were elongated, with moderate intensity profiles, while cell-associated virions were compact and very bright (Fig. 2C). The reconstructions were in agreement with mimicry-embedded virions, suggesting that these properties yielded the base for the high classification accuracy (Fig. 2D).

Given its ability to improve CapsNet-based classification of our data set, we wondered if mimicry embedding would also enhance the performance of other neural network architectures with increasing expressive capacity (Table 1). To this end, using a conventional feed-forward network (1,570 trainable parameters) as a baseline, we compared LeNet CNN (30,472 trainable parameters) (7), ResNet-101 (42,542,978 trainable parameters) (27), and CapsNet (7,281,680 trainable parameters). We found that mimicry embedding did not improve the performance of low-expressive-capacity architectures, such as the small feed-forward network, but it has a tendency to improve the performance of high-expressive-capacity architectures on small data sets (Table 1). Furthermore, in the case of CapsNet architecture, it is likely that the random initialization of weights caused the issue with our VACV data set, as it is small and complex, two things CapsNet struggles with (18).

**TABLE 1:** Comparison of mimicry-embedding performance applied to various neural network architectures^*a*^

Condition	Precision	Recall	F1 score	AUC
Feed forward: data set	0.96	0.96	0.96	0.98
Feed forward: mimicry-embedded data set	0.96	0.96	0.96	0.98
LeNet: data set	0.79	0.79	0.76	0.87
LeNet: mimicry-embedded data set	0.81	0.81	0.79	0.89
ResNet-101: data set	0.92	0.92	0.92	0.96
ResNet-34: mimicry-embedded data set	0.96	0.96	0.96	0.98
CapsNet: data set				
CapsNet: mimicry-embedded data set	0.96	0.96	0.96	0.98

a AUC, area under the receiver operating characteristics curve. All metrics are averaged as one-versus-rest across classes. A missing value indicates “no convergence.”

**FIG 3:**
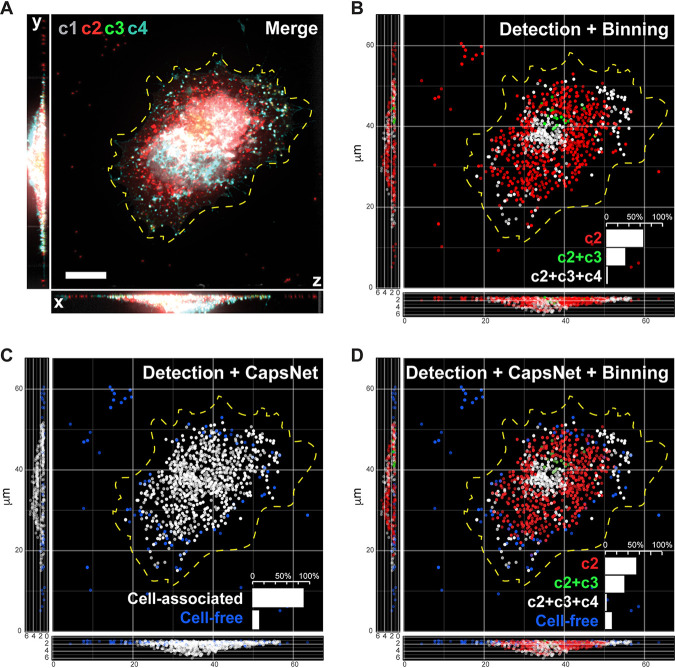
Inference demonstrates that mimicry embedding and trained CapsNet allow efficient classification of VACV particles into four biological classes. (A) Merged four-channel fluorescent image of a HeLa cell infected with VACV previously unseen by CapsNet (see Fig. S1A for channel details). Bar, 10 μm. (B) Respective ZedMate particle detection and classification by conventional binning of fluorescence intensities. (C) Respective inference of cell-free and cell-associated particles detected by ZedMate, mimicry embedded and predicted by a trained CapsNet (Fig. 2B and C). (D) Combined ZedMate particle detection with mimicry-embedded and trained CapsNet results in classification of four types of biologically relevant VACV particles. (Insets) Quantification of the particle types in the respective image. Statistical validation of machine learning models is provided in Fig. S3.

To verify the impact of mimicry embedding on CapsNet, we performed inference on an unseen (separate from training and validation sets) experimental data set. Figure 3 shows the workflow, from an input four-channel image (Fig. 3A and Fig. S1A and B) to detection and binning of virions (Fig. 3B), followed by mimicry embedding and CapsNet separation of cell-free versus cell-associated virions (Fig. 3C). The results indicate that our model and binning combined allow accurate classification of virions into four biologically relevant classes within unseen data sets (Fig. 3D).

**FIG 4:**
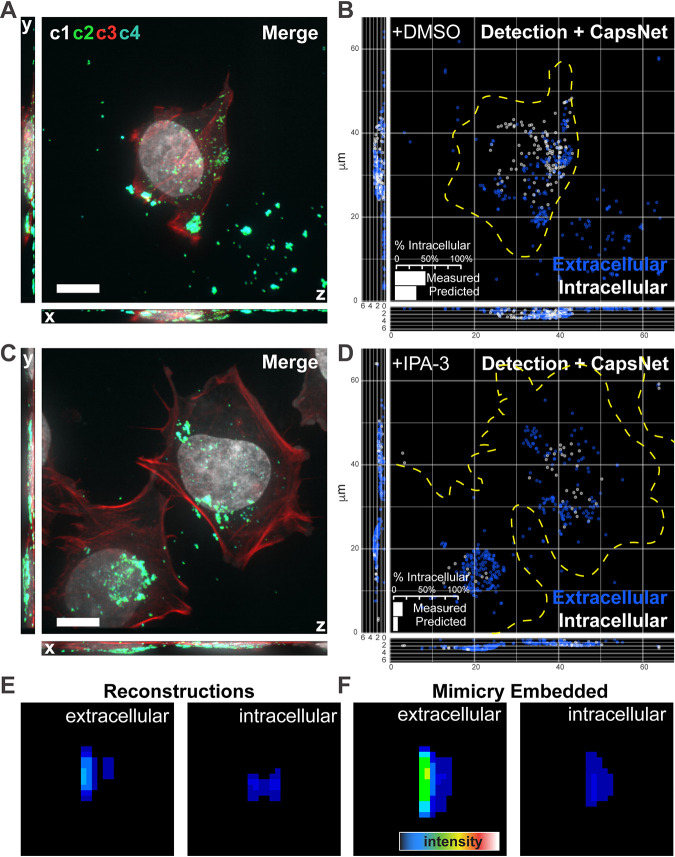
Mimicry embedding can be used for weak-labeling particle classification. (A) Merged four channel fluorescent image of a HeLa cell infected with VACV previously unseen by CapsNet (see Fig. S4A for channel details). (B) ZedMate detection and trained-CapsNet-predicted extracellular and intracellular particles. (Inset) Quantification of intracellular particles. (C) Merged four-channel image of HeLa cell infected with VACV and treated with the entry inhibitor IPA-3, previously unseen by CapsNet. (D) ZedMate detection and trained CapsNet inference of intracellular and extracellular particles. (Inset) Quantification of intracellular particles. (E) Representative reconstruction profiles of extra- and intracellular virions. (F) Representative mimicry-embedded extra- and intracellular VACV particles for comparison to the images in panel E. *n* = 40 (3 biological replicates) untreated and treated cells each. Detailed model performance (statistical validation) metrics are provided in Fig. S4. Bars, 10 μm.

We have established that mimicry embedding and weights transfer allows us to distinguish between incoming cell-free and newly assembled cell-associated virions at late time points after infection. Next, we asked if this approach could also be used to classify extracellular versus intracellular virions during virus entry, a single-cell assay that often requires specific antibodies or labeling strategies and labor-intensive manual annotation (see “Superresolution imaging of VACV intracellular virions” in Materials and Methods). Considering these common limitations, we generated a training data set consisting of 30,537 images that would allow generalization of this approach. Early infected cells, with virions seen in c2, were stained with common fluorescent DNA (c1) and actin (c3) dyes. To circumvent hand-labeling of the training data, immunolabeling to distinguish between intra- and extracellular virus (c4) was used as a weak labeling (28) strategy (Fig. 4A and Fig. S4A). After ZedMate detection and transformation of individual particles, intra- and extracellular virus weak labeling (c4), which distinguished 21,566 extracellular and 8,971 intracellular virions, was removed for mimicry embedding. By maintaining our binary MNIST-trained CapsNet and performing weights transfer, we could achieve 82% accuracy (81.3% precision, 81.4% recall) in differentiating between intra- and extracellular virions in the absence of specific-antibody labeling and manual annotation (See Fig. S4B to E for classifier training).

10.1128/mSphere.00836-20.4 FIG S4 CapsNet training and validation of extracellular versus intracellular virus model. (A) Maximum-intensity projections of individual channels from HeLa cells infected with VACV. Channles were DNA stain (c1), VACV core A5-EGFP (c2), actin stained with phalloidin (c3) and VACV membrane protein L1 as an extracellular virion label (c4). (B) Model loss function change upon training iterations (epochs). (C) Model training and validation (unseen data) accuracy change upon training iterations (epochs). (D) Model receiver operational characteristics (ROC) curve of the trained model obtained using unseen data (validation). Here, the area under the curve (AUC) was 0.896. (E) Model confusion matrix of the trained model obtained using unseen data (validation). Model precision was 81.3%, recall was 81.4%, and the F1 score was 81.8%. Download FIG S4, TIF file, 0.6 MB. Copyright © 2020 Yakimovich et al. 2020 Yakimovich et al. This content is distributed under the terms of the Creative Commons Attribution 4.0 International license.

To estimate accuracy, inference was performed on an unseen data set in which intra- and extracellular virions were quantified using c1 to c4 (measured), inclusive of extracellular virion weak labeling, or only c1 to c3 (predicted) (Fig. 4B). An 86% match between measured and predicted quantification of intracellular particles was seen (Fig. 4B, inset). This indicates that weak labeling can effectively substitute for manual annotation of training data sets when intra- and extracellular virion signals are being classified. As an additional test of the ANN, we generated a data set skewed for extracellular virions by blocking virus entry with IPA-3 (29, 30) (Fig. 4C). Consistent with its performance (Fig. S4B to E), a 93% match between measured and predicted quantifications of intracellular particles was seen (Fig. 4D). Finally, when we visualized the reconstructions of intra- and extracellular virion classes, extracellular virions appeared brighter and more elongated in the Z direction than intracellular ones (Fig. 4E). This was in agreement with their mimicry-embedded counterparts (Fig. 4F), explaining the ANNs’ ability to accurately predict between and quantify these two virion classes.

### Mimicry embedding enables deep neural network training for Toxoplasma gondii viability analysis.

**FIG 5:**
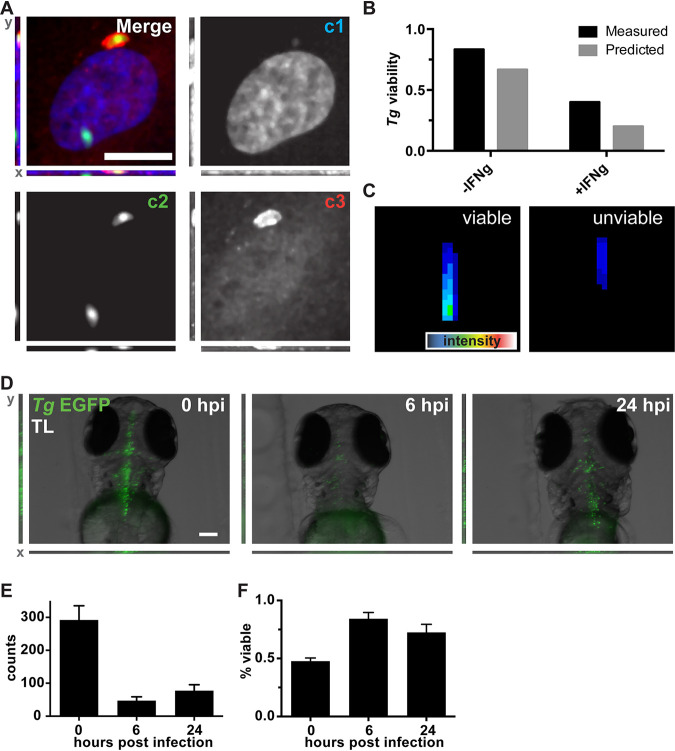
Mimicry embedding and weight transfer employed for Toxoplasma gondii (*Tg*) viability detection in cell culture and *in vivo*. (A) Merged three-channel fluorescent image of a HUVEC infected with T. gondii-EGFP. Individual channels represent DNA stain (c1), T. gondii-EGFP (c2), and ubiquitin (c3). A total of 2,694 images were obtained from 3 biological replicates. Bar, 25 μm. (B) Quantification of weakly labeled (measured) and CapsNet-inferred (predicted) viable and unviable parasites. (C) Representative reconstructions of the trained CapsNet network for viable and unviable classes of T. gondii-EGFP Z-profiles. (D) Representative images (maximum-intensity projections) of zebrafish (*D. rerio*) larvae infected with T. gondii-EGFP at 0, 6, and 24 hpi. The same 10 larvae were followed over time. Bar, 100 μm. (E) ZedMate-detected T. gondii counts at 0, 6, and 24 h postinfection. (F) *In vivo* inference of T. gondii-EGFP viability over time using the DropConnect viability model trained on *in vitro* T. gondii data. *n* = 10 Z-stack images per time point (3 biological replicates). Values are means and SEM. Statistical validation of machine learning models is provided in Fig. S5.

To assess the general applicability of our mimicry embedding approach, we acquired an imaging data set of cells infected with an enhanced green fluorescent protein (EGFP)-expressing version of the parasite Toxoplasma gondii. While T. gondii*-*EGFP is readily visualized by conventional microscopy, detecting and quantifying intracellular viability at the single parasite level are challenging (14). To generate a T. gondii viability training data set, cells infected with T. gondii-EGFP (c1) were fixed and stained with fluorescent markers of DNA (c2) and host cell ubiquitin (c3), which was used as a weak label to annotate the subset of “unviable” parasites (14, 31) (Fig. 5A). Individual particle detection and transformation in ZedMate were followed by mimicry embedding in the absence of c3 weak labeling. This training data set, generated through random sampling, was composed of 2,694 images containing equal proportions of viable and unviable examples. After weights transfer from the CapsNet trained on binary MNIST (Fig. 2B) and fine tuning on T. gondii-EGFP, we achieved 70% accuracy (precision, 72.5%; recall, 70.7%) in the absence of specific viability labeling (see Fig. S5A to D for classifier training).

10.1128/mSphere.00836-20.5 FIG S5 CapsNet and DropConnect training and validation of in vitro and in vivo T. gondii-EGFP viability model. (A) Two-channel CapsNet model loss function change upon training iterations (epochs). (B) Two-channel CapsNet model training and validation (unseen data) accuracy change upon training iterations (epochs). (C) Two-channel CapsNet model receiver operational characteristics (ROC) curve of the trained model obtained using unseen data (validation). Here, the area under the curve (AUC) was 0.764. (D) Two-channel CapsNet model confusion matrix of the trained model obtained using unseen data (validation). (E) One-channel DropConnect model loss function change upon training iterations (epochs). (F) One-channel DropConnect model training and validation (unseen data) accuracy change upon training iterations (epochs). (G) One-channel DropConnect model receiver operational characteristics (ROC) curve of the trained model obtained using unseen data (validation). Here, the area under the curve (AUC) was 0.685. (H) One-channel DropConnect model confusion matrix of the trained model obtained using unseen data (validation). The 2-channel CapsNet model precision was 72.5%, recall was 70.7%, and the F1 score was 70.1%. The 1-channel DropConnect model precision was 65.9%, recall was 64.3%, and the F1 score was 64.9%. Download FIG S5, TIF file, 0.3 MB. Copyright © 2020 Yakimovich et al. 2020 Yakimovich et al. This content is distributed under the terms of the Creative Commons Attribution 4.0 International license.

To ensure that the ANN could accurately distinguish between viable and unviable parasites, we generated a data set of cells infected with T. gondii-EGFP using a specific viability label (c3) as ground truth (Fig. 5A). To further assess viability, experiments were performed in the absence or presence of gamma interferon (IFN-γ), which drives parasite killing. Upon model training and validation, test inference on this data set using c1-c2 resulted in 84% and 80% matches between measured (c3) and predicted (c1-c2) viability in the absence or presence of IFN-γ, respectively (Fig. 5B). CapsNet generator reconstructions showed that “viable” T. gondii-EGFP organisms appear larger and brighter than “unviable” parasites in both c1 and c2 (Fig. 5C). This likely explains the ability of the model to accurately predict T. gondii-EGFP viability in the absence of specific c3 viability labeling.

In an attempt to train a general model for *in vivo* parasite viability assessment using our *in vitro* data set, we performed mimicry embedding on T. gondii-EGFP (Fig. 5A, c2 panel). This resulted in a >10% drop in prediction accuracy when training on CapsNet. This suggested that single-channel mimicry embedding does not provide enough context for training of complex algorithms. However, we reasoned that as our mimicry embedding is based on MNIST, we could use any algorithm that performs well on this data set. By switching to DropConnect (32) architecture, which performs among the best on MNIST, our classifier achieved 65% accuracy (precision, 65.9%; recall, 64.3%) in differentiating between viable and unviable parasites using a single channel (see Fig. S5E to H for classifier training).

To test this classifier on an *in vivo* data set, we infected zebrafish (Danio rerio) larvae with T. gondii-EGFP in the hindbrain ventricle. Infected larvae were imaged at 0, 6, and 24 h after infection by fluorescent 3D stereomicroscopy (Fig. 5D). ZedMate was used to detect and quantify T. gondii-EGFP numbers over time (Fig. 5E). A dramatic drop in parasite count was seen between infection at 0 h and 6 h, followed by increased numbers of T. gondii-EGFP by 24 h. Next, the T. gondii-EGFP Z-profiles were mimicry embedded and normalized, and viability was inferred using the *in vitro* infected-cell model previously trained on DropConnect. At high pathogen load (0 h), 48% of T. gondii-EGFP organisms were scored as viable (Fig. 5F). By 6 h, this increased to 95%, and there was no significant change within 24 h. These results are consistent with an initial clearing of unviable parasites between 0 and 6 h and replication of the remaining viable ones (33).

**FIG 6:**
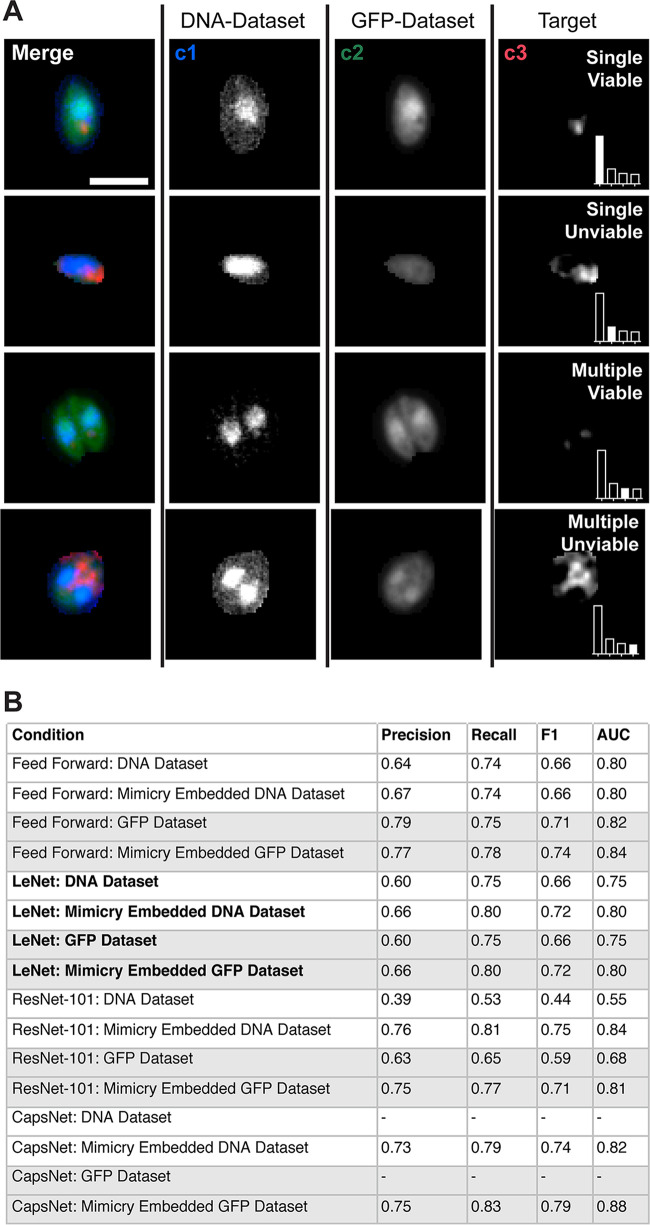
2D Toxoplasma gondii vacuole data set formulated as a multiclass classification problem. Maximum-intensity projections of merged and individual fluorescence channels of T. gondii EGFP vacuoles. Individual channels represent DNA stain (c1; DNA-Dataset), T. gondii EGFP (c2; GFP-Dataset), and ubiquitin (c3), which was used to obtain target information on viability. Bar, 5 μm. (Insets) Bar plots demonstrate proportions of number of examples in classes; filled bars represent the current class. (B) Impact of mimicry embedding on 2D data sets across various neural network architectures. AUC, area under the receiver operating characteristics curve. All metrics are averaged as one-versus-rest across classes.

Finally, we sought to determine the utility of mimicry embedding for analysis of 2D data sets. To this end, we generated a 3-channel, multiclass 2D T. gondii-EGFP vacuole data set using maximum intensity projections (MIPs) of high-content microscopy images. For this, T. gondii-EGFP-containing vacuoles were detected and cropped into individual images. The vacuole MIPs were split into DNA (c1), EGFP (c2), and host cell ubiquitin (c3) channels. Here, c3 was used as a weak label to annotate the subset of “unviable” parasites, thereby defining the ground truth for parasite viability (14, 31). Lastly, vacuole images were sorted into single- and multiple-pathogen subclasses, resulting in a complex 2D data set of 7,021 images annotated into 4 classes: single viable (3,922 images), single unviable (1,510 images), multiple viable (840 images), and multiple unviable (749 images) (Fig. 6A).

Using this data set, we compared the performance of various architectures with and without mimicry embedding (Fig. 6B). As with low-complexity binary data sets (Table 1), mimicry embedding did not enhance the performance on either c1 or c2 data sets with the baseline low-expressive-capacity feed-forward network. However, it did provide marked improvement in both c1 and c2 when networks with high expressive capacity were used. This was most pronounced in the case of ResNet-101, which performed poorly when confronted with four classes versus two, and CapsNet, which again failed to initialize. These results support our conclusion that mimicry embedding is useful for higher-expressive-capacity architectures, like ResNet and CapsNet, when training on small and unbalanced data sets, such as those often found in biomedical imaging.

## DISCUSSION

ANN analysis of fluorescent host-pathogen data sets has trailed behind the unprecedented advancement of artificial intelligence (AI) analysis seen in other fields. This is largely due to the lack of open-source, verified pathogen data sets comparable to MNIST and ImageNet (20, 21). Here, we present ZedMate and mimicry embedding as a strategy to harness the power of verified data sets like MNIST and transfer learning to train highly accurate models for analysis of biomedical images.

Other strategies that aim to improve the performance of deep neural networks on real-world data sets include image augmentation (15, 34), self-supervised learning (35, 36) and semisupervised learning (37). Image augmentation aims to boost the size of the data set by applying linear changes to it. While routinely used in deep learning systems, only limited augmentation types remain sensible for strictly controlled domains like microscopy. Both semisupervised and self-supervised approaches relieve the need for labeled data but do not eliminate the requirement for large unlabeled data sets. This leaves a void in the biomedical image analysis domain, where often only shallow learning approaches (e.g., feed-forward networks) that do not fully appreciate the phenotypic complexity in biology are applicable. Mimicry embedding aims to fill this void, allowing researchers to utilize large-expressive-capacity models capable of learning complex data representations. While the representations learned from an unrelated verified data set, like MNIST, might not be optimal for all scenarios, our results show that this may prove useful for network initialization.

By testing a variety of neural network architectures of increasing expressive capacity, we found that mimicry embedding does not change the ability of low-expressive-capacity feed-forward networks but improves the performance of high-expressive-capacity neural networks, including ResNet and CapsNet. This intuitively suggests that mimicry embedding works through lowering the data set size requirement for high-capacity architectures, perhaps through including the representations learned from the standardized data set.

In addition, we show that mimicry embedding works on both 2D and, in conjunction with ZedMate, 3D images. We noted that for LeNet and ResNet, mimicry embedding proved more useful for multiclass, as opposed to binary, classification problems. The possible explanation for this is that, compared to binary classification problems, multiclass classification problems may elevate the requirements for the data set size even for shallower networks, like LeNet.

As such, mimicry embedding is a valuable addition to the data science arsenal. When used together with CapsNet (16), LeNet, and ResNet, mimicry embedding proved to be a promising method for detection of complex pathogen phenotypes *in vitro*. We show that transforming real-world images such that they resemble landmark data sets ensures compatibility with, and seamless switching between, cutting-edge architectures. Embedding data in such a way allows one to maintain full compatibility with weights of the first layers, thereby improving transfer. Using *in vivo* pathogen data, we further demonstrate that mimicry embedding can yield a model with higher accuracy than one obtained through cutting-edge neural architecture search. Collectively, our results suggest that ZedMate and mimicry embedding, although employed here for the analysis of host-pathogen interaction, can be useful for AI analysis of small unbalanced 2D and 3D fluorescent data sets.

## MATERIALS AND METHODS

### Cell culture, antibodies, and reagents.

HeLa cells (ATCC) were maintained in in Dulbecco's modified Eagle's medium (DMEM; Gibco, Life Technologies, Switzerland) with the addition of 10% fetal bovine serum (FBS; Sigma), 1% penicillin-streptomycin (Pen-Strep; Sigma), and 2 mM GlutaMAX (Life Technologies). Human umbilical vein endothelial cells (HUVECs; C12203; Promocell) were maintained in M199 medium (Gibco) supplemented with 30 mg/ml endothelial cell growth supplement (ECGS; 02–102; Upstate), 10 units/ml heparin (H-3149; Sigma), and 20% FBS (Sigma). Cells were cultivated on plates precoated with 1% (wt/vol) porcine gelatin (G1890; Sigma). Both HUVECs and HeLa cells were grown as monolayers at 37.0°C and 5.0% CO_2_. HUVECs were not used beyond passage 6.

Hoechst 33342 (Sigma) was used postfixation at a 1:10,000 dilution throughout. Cell culture-grade dimethyl sulfoxide (DMSO), used to dissolve control experimental compounds, was obtained from Sigma.

### VACV strain and virus purification.

Vaccinia virus strain Western Reserve expressing A5 mCherry protein (VACV WR) was used throughout (29, 38, 39). VACV mature virions (MVs) were purified from cytoplasmic lysates by being pelleted through a 36% sucrose cushion for 90 min at 18,000 × *g*. The virus pellet was resuspended in 10 mM Tris (pH 9.0) and subsequently banded on a 25% to 40% sucrose gradient at 14,000 × *g* for 45 min. Following centrifugation, the viral band was collected by aspiration and concentrated by pelleting at 14,000 × *g* for 45 min. MVs were resuspended in 1 mM Tris (pH 9.0), and the titer was determined as PFU per milliliter as previously described (40).

### Early VACV infection and extracellular virion staining.

HeLa cells were seeded onto CELLview slides (Greiner Bio-One) at 10,000 cells per well 16 h before the experiment. VACV A5-mCherry F13-EGFP was added at a multiplicity of infection (MOI) of 20, to increase the chances of synchronous infection. Cells were fixed with 4% electron microscopy (EM)-grade paraformaldehyde (PFA) 4 h postinfection (hpi) for 20 min followed by a phosphate-buffered saline (PBS) wash. Staining and labeling were preceded by blocking (without permeabilization) in blocking buffer (5% bovine serum albumin [BSA]–1% FBS in PBS) for 60 min at room temperature (RT). Next, L1 mouse (7D11) antibody (41) (1:1,000) in blocking buffer was added for 60 min at RT, followed by a PBS wash. Anti-mouse antibody (Alexa Fluor 647; 1:1,000; A32728; Invitrogen), phalloidin 594 (1:1,000; Sigma) and Hoechst in blocking buffer were added for 60 min at RT, followed by a PBS wash. 1,1′-Disulfanediyldinaphthalen-2-ol VACV entry inhibitor (IPA-3) was obtained and used as described elsewhere (38). The DMSO concentration was equal to or below 1%.

### Late VACV infection and staining.

HeLa cells were cultured on coverslips and infected with VACV WR expressing A5 mCherry protein. At 8 hpi, cells were fixed with 4% (vol/vol) FA. Next, VACV B5 protein antibody (mouse; 1:1,000) in blocking buffer was added for 60 min at RT, followed by a PBS wash. Anti-mouse antibody (Alexa Fluor 647; A32728; Invitrogen) and Hoechst in blocking buffer were added for 60 min at RT, followed by a PBS wash.

### Toxoplasma gondii cultivation.

*Toxoplasma* (RH type I and Prugniaud type II strains) expressing GFP or luciferase was maintained *in vitro* by serial passage on human foreskin fibroblast (HFF) cultures (ATCC). Cultures were grown in high-glucose DMEM (Life Technologies) supplemented with 10% FBS (Life Technologies) at 37°C in 5% CO_2_.

### T. gondii cultured cell infection and staining.

The day before the infection, type II parasites were passaged onto new HFFs to obtain parasites with a high viability. T. gondii were prepared from HFF cultures that had been freshly syringe lysed with a 25-gauge syringe. Parasites were subsequently syringe lysed twice with a 27-gauge syringe, and excess HFF cell debris was removed by centrifugation. Then, the parasites were added to the experimental cells at an MOI of 2. The cell cultures with added T. gondii were then centrifuged at 500 × *g* for 5 min to synchronize the infection, and the cultures were incubated at 37°C and 5% CO_2_ for 3 h. Samples treated with IFN-γ were subjected to 100 IU/ml human IFN-γ (285-IF; R&D Systems) for 18 h prior to infection. Upon fixation, cells were stained with Hoechst 33342 and mouse anti-ubiquitin monoclonal antibody (MAb) FK2 (PW8810; Enzo Lifesciences; RRID AB_10541840) and Alexa Fluor 568-conjugated secondary goat anti-mouse antibody (A-11004; Invitrogen; RRID AB_141371).

### T. gondii infection *in vivo*.

T. gondii-EGFP parasites (type 1) were prepared from freshly 25-gauge-syringe-lysed HFF cultures in 10% FBS. Parasites were subsequently 27-gauge syringe lysed, and excess HFF material was removed by centrifugation. After a washing with PBS, *Toxoplasma* tachyzoites were resuspended at 2.0 × 10^6^ tachyzoites/μl in PBS.

Zebrafish larvae were anesthetized with 20 μg/ml tricaine (Sigma-Aldrich) during the injection procedures and for all live *in vivo* imaging. All experiments were carried out on TraNac-background larvae to minimize obstruction of fluorescence signal by pigmentation (42). A 3 days postfertilization (dpf), 10 larvae were anesthetized and injected with ∼2.0 nl of parasite suspension into the hindbrain ventricle (HBV) as previously described (43). Infected larvae were transferred into individual wells containing 0.5× E2 medium supplemented with methylene blue and prewarmed to 33°C. No infection-mediated mortality was observed.

### Zebrafish husbandry and maintenance.

Fish were maintained at 28.5°C on a 14-h light, 10-h dark cycle. Embryos obtained by natural spawning were maintained in 0.5× E2 medium supplemented with 0.3 μg/ml methylene blue.

### Ethics statement.

Animal experiments were performed according to the Animals (Scientific Procedures) Act of 1986 and approved by the Home Office (project licenses PPL P84A89400 and P4E664E3C). All experiments were conducted up to 4 days postfertilization.

### Superresolution imaging of VACV intracellular virions.

Superresolution microscopy was performed using a 100× oil immersion objective (numerical aperture [NA], 1.45) on a VT-iSIM microscope (Visitech; Nikon Eclipse TI), using 405-nm, 488-nm, 561-nm, and 647-nm laser frequencies for excitation.

### High-content T. gondii EGFP imaging in cells.

Black plastic flat-bottom 96-well plates (Falcon number 353219) were imaged on an Opera Phenix high-content imaging platform using ×63 magnification, 8 Z-slices (0.5 μm/slice), and multiple fields of view per well. Images were saved as single channel 16-bit TIFF files and further processed for ZedMate analysis.

### 3D T. gondii-EGFP imaging *in vivo*.

Progress of the *in vivo* infection was monitored by fluorescent stereomicroscopy (Leica M205FA; Leica Microsystems, Nussloch GmbH, Nussloch, Germany) at regular time points. The same 10 larvae were followed over time. All images were obtained with a 1× objective, at ×130 magnification (0.79 μm/pixel). Twenty Z-planes were captured covering a total distance of 171 μm (8.55-μm intervals). All image analysis and quantification were automated and carried out using ZedMate, thus circumventing the need for sample randomization during image analysis.

### Data processing and deep neural network training.

Our training hardware was based on a single Nvidia 1080 Ti GPU set up in an Intel Core i7 8700K system equipped with 32 Gb of RAM and an SSD. Installation consisted of Anaconda Python, Keras-gpu 2.2, Tensorflow-gpu 1.10 and KNIME 3.7.1. Some models were trained on 2019 MacBook Pro equipped with Intel Core i5 CPU using a Keras 2.2 CPU.

### Data availability.

The data sets generated and/or analyzed during the current study are available from the corresponding authors on request. Source code is available at https://github.com/ayakimovich/ZedMate, and an example data set can be found at https://github.com/ayakimovich/virus-mnist.

## References

[1] Collobert R, Weston J. 2008. A unified architecture for natural language processing: deep neural networks with multitask learning, p 160–167. *In* ICML ’08: the 25th Annual International Conference on Machine Learning. ACM, New York, NY.

[2] Schroff F, Kalenichenko D, Philbin J. 2015. Facenet: a unified embedding for face recognition and clustering, p 815–823. *In* 2015 IEEE Conference on Computer Vision and Pattern Recognition. IEEE, New York, NY.

[3] Ramos S, Gehrig S, Pinggera P, Franke U, Rother C. 2017. Detecting unexpected obstacles for self-driving cars: fusing deep learning and geometric modeling, p 1025–1032. *In* 2017 IEEE Intelligent Vehicles Symposium. IEEE, New York, NY.

[4] Kim J, Canny J. 2017. Interpretable learning for self-driving cars by visualizing causal attention, p 2942–2950. *In* 2017 IEEE International Conference on Computer Vision. IEEE, New York, NY.

[5] LeCun Y, Bengio Y. 1995. Convolutional networks for images, speech, and time series, p 255–258. *In* Arbib MA (ed), The handbook of brain theory and neural networks. MIT Press, Cambridge, MA.

[6] LeCun Y, Bengio Y, Hinton G. 2015. Deep learning. Nature 521:436–444. doi:10.1038/nature14539. 26017442

[7] LeCun Y, Bottou L, Bengio Y, Haffner P. 1998. Gradient-based learning applied to document recognition. Proc IEEE 86:2278–2324. doi:10.1109/5.726791.

[8] Angermueller C, Pärnamaa T, Parts L, Stegle O. 2016. Deep learning for computational biology. Mol Syst Biol 12:878. doi:10.15252/msb.20156651. 27474269 PMC4965871

[9] Wei JN, Duvenaud D, Aspuru-Guzik A. 2016. Neural networks for the prediction of organic chemistry reactions. ACS Cent Sci 2:725–732. doi:10.1021/acscentsci.6b00219. 27800555 PMC5084081

[10] Segler MH, Waller MP. 2017. Neural‐symbolic machine learning for retrosynthesis and reaction prediction. Chemistry 23:5966–5971. doi:10.1002/chem.201605499. 28134452

[11] Weigert M, Schmidt U, Boothe T, Müller A, Dibrov A, Jain A, Wilhelm B, Schmidt D, Broaddus C, Culley S, Rocha-Martins M, Segovia-Miranda F, Norden C, Henriques R, Zerial M, Solimena M, Rink J, Tomancak P, Royer L, Jug F, Myers EW. 2018. Content-aware image restoration: pushing the limits of fluorescence microscopy. Nat Methods 15:1090–1097. doi:10.1038/s41592-018-0216-7. 30478326

[12] Jug F, Pietzsch T, Preibisch S, Tomancak P. 2014. Bioimage informatics in the context of Drosophila research. Methods 68:60–73. doi:10.1016/j.ymeth.2014.04.004. 24732429

[13] Caicedo JC, Cooper S, Heigwer F, Warchal S, Qiu P, Molnar C, Vasilevich AS, Barry JD, Bansal HS, Kraus O, Wawer M, Paavolainen L, Herrmann MD, Rohban M, Hung J, Hennig H, Concannon J, Smith I, Clemons PA, Singh S, Rees P, Horvath P, Linington RG, Carpenter AE. 2017. Data-analysis strategies for image-based cell profiling. Nat Methods 14:849–863. doi:10.1038/nmeth.4397. 28858338 PMC6871000

[14] Fisch D, Yakimovich A, Clough B, Wright J, Bunyan M, Howell M, Mercer J, Frickel E. 2019. Defining host–pathogen interactions employing an artificial intelligence workflow. Elife 8:e40560. doi:10.7554/eLife.40560. 30744806 PMC6372283

[15] Krizhevsky A, Sutskever I, Hinton GE. 2012. Imagenet classification with deep convolutional neural networks. Adv Neural Inf Process Systems 25:1097–1105.

[16] Sabour S, Frosst N, Hinton GE. 2017. Dynamic routing between capsules, p 3856–3866. *In* Advances in neural information processing systems.

[17] Xi E, Bing S, Jin Y. 2017. Capsule network performance on complex data. https://arxiv.org/abs/1712.03480.

[18] Mukhometzianov R, Carrillo J. 2018. CapsNet comparative performance evaluation for image classification. https://arxiv.org/abs/1805.11195.

[19] Ronneberger O, Fischer P, Brox T. 2015. U-net: convolutional networks for biomedical image segmentationn, p 234–241. *In* International conference on medical image computing and computer-assisted intervention. Springer, Cham, Switzerland.

[20] Deng L. 2012. The MNIST database of handwritten digit images for machine learning research [best of the web]. IEEE Signal Processing Magazine 29:141–142. doi:10.1109/MSP.2012.2211477.

[21] Deng J, Dong W, Socher R, Li L-J, Li K, Fei-Fei L. 2009. ImageNet: a large-scale hierarchical image database, p 248–255. *In* 2009 IEEE Conference on Computer Vision and Pattern Recognition, Miami, FL, 2009. IEEE, New York, NY.

[22] West J, Ventura D, Warnick S. 2007. A theoretical foundation for inductive transfer. Brigham Young University, College of Physical and Mathematical Sciences.

[23] Pan SJ, Yang Q. 2010. A survey on transfer learning. IEEE Trans Knowl Data Eng 22:1345–1359. doi:10.1109/TKDE.2009.191.

[24] Usaj MM, Styles EB, Verster AJ, Friesen H, Boone C, Andrews BJ. 2016. High-content screening for quantitative cell biology. Trends Cell Biol 26:598–611. doi:10.1016/j.tcb.2016.03.008. 27118708

[25] Schindelin J, Arganda-Carreras I, Frise E, Kaynig V, Longair M, Pietzsch T, Preibisch S, Rueden C, Saalfeld S, Schmid B, Tinevez J-Y, White DJ, Hartenstein V, Eliceiri K, Tomancak P, Cardona A. 2012. Fiji: an open-source platform for biological-image analysis. Nat Methods 9:676–682. doi:10.1038/nmeth.2019. 22743772 PMC3855844

[26] Tinevez J-Y, Perry N, Schindelin J, Hoopes GM, Reynolds GD, Laplantine E, Bednarek SY, Shorte SL, Eliceiri KW. 2017. TrackMate: an open and extensible platform for single-particle tracking. Methods 115:80–90. doi:10.1016/j.ymeth.2016.09.016. 27713081

[27] He K, Zhang X, Ren S, Sun J. 2016. Deep residual learning for image recognition, p 770–778. *In* 2016 IEEE Conference on Computer Vision and Pattern Recognition, Las Vegas, NV, 2016. IEEE, New York, NY.

[28] Ratner AJ, De Sa CM, Wu S, Selsam D, Ré C. 2016. Data programming: creating large training sets, quickly. Adv Neural Inf Process Syst 29:3567–3575. 29872252 PMC5985238

[29] Mercer J, Helenius A. 2008. Vaccinia virus uses macropinocytosis and apoptotic mimicry to enter host cells. Science 320:531–535. doi:10.1126/science.1155164. 18436786

[30] Deacon SW, Beeser A, Fukui JA, Rennefahrt UE, Myers C, Chernoff J, Peterson JR. 2008. An isoform-selective, small-molecule inhibitor targets the autoregulatory mechanism of p21-activated kinase. Chem Biol 15:322–331. doi:10.1016/j.chembiol.2008.03.005. 18420139 PMC4353635

[31] Clough B, Wright JD, Pereira PM, Hirst EM, Johnston AC, Henriques R, Frickel E-M. 2016. K63-linked ubiquitination targets Toxoplasma gondii for endo-lysosomal destruction in IFNγ-stimulated human cells. PLoS Pathog 12:e1006027. doi:10.1371/journal.ppat.1006027. 27875583 PMC5119857

[32] Wan L, Zeiler M, Zhang S, Le Cun Y, Fergus R. 2013. Regularization of neural networks using DropConnect. Proc Machine Learning Res 28:1058–1066.

[33] Yoshida N, Domart M-C, Peddie CJ, Yakimovich A, Mazon-Moya MJ, Hawkins TA, Collinson L, Mercer J, Frickel E-M, Mostowy S. 2020. The zebrafish as a novel model for the *in vivo* study of *Toxoplasma gondii* replication and interaction with macrophages. Dis Models Mech 13:dmm043091. doi:10.1242/dmm.043091. PMC7390642 32461265

[34] Shorten C, Khoshgoftaar TM. 2019. A survey on image data augmentation for deep learning. J Big Data 6:60. doi:10.1186/s40537-019-0197-0. PMC8287113 34306963

[35] Korbar B, Tran D, Torresani L. 2018. Cooperative learning of audio and video models from self-supervised synchronization, p 7774–7785. *In* NIPS’18: Proceedings of the 32nd International Conference on Neural Information Processing Systems. ACM, New York, NY.

[36] Mahendran A, Thewlis J, Vedaldi A. 2019. Cross pixel optical-flow similarity for self-supervised learning, p 99–116. *In* Jawahar C, Li H, Mori G, Schindler K (ed), Computer vision–ACCV 2018. Lecture notes in computer science, vol 11365. Springer, Cham, Switzerland.

[37] Chapelle O, Schölkopf B, Zien A. 2006. A discussion of semi-supervised learning and transduction, p 473–478. *In* Chapelle O, Scholkopf B, Zien A (ed), Semi-supervised learning. MIT Press, Cambridge, MA.

[38] Mercer J, Knebel S, Schmidt FI, Crouse J, Burkard C, Helenius A. 2010. Vaccinia virus strains use distinct forms of macropinocytosis for host-cell entry. Proc Natl Acad Sci U S A 107:9346–9351. doi:10.1073/pnas.1004618107. 20439710 PMC2889119

[39] Kilcher S, Mercer J. 2015. DNA virus uncoating. Virology 479–480:578–590. doi:10.1016/j.virol.2015.01.024. 25728300

[40] Mercer J, Traktman P. 2003. Investigation of structural and functional motifs within the vaccinia virus A14 phosphoprotein, an essential component of the virion membrane. J Virol 77:8857–8871. doi:10.1128/jvi.77.16.8857-8871.2003. 12885904 PMC167248

[41] Wolffe EJ, Vijaya S, Moss B. 1995. A myristylated membrane protein encoded by the vaccinia virus L1R open reading frame is the target of potent neutralizing monoclonal antibodies. Virology 211:53–63. doi:10.1006/viro.1995.1378. 7645236

[42] Krauss J, Astrinidis P, Astrinides P, Frohnhöfer HG, Walderich B, Nüsslein-Volhard C. 2013. transparent, a gene affecting stripe formation in zebrafish, encodes the mitochondrial protein Mpv17 that is required for iridophore survival. Biol Open 2:703–710. doi:10.1242/bio.20135132. 23862018 PMC3711038

[43] Yoshida N, Domart M-C, Peddie CJ, Yakimovich A, Mazon-Moya MJ, Hawkins TA, Collinson L, Mercer J, Frickel E-M, Mostowy S. 2020. The zebrafish as a novel model for the in vivo study of Toxoplasma gondii replication and interaction with macrophages. Dis Model Mech 13:dmm043091. doi:10.1242/dmm.043091. 32461265 PMC7390642

