# Supplementary figures and images for: Mimicry Embedding Facilitates Advanced Neural Network Training for Image-Based Pathogen Detection
Source: mSphere. 2020 Sep 9;5(5):e00836-20. doi: 10.1128/mSphere.00836-20 (PMC7485691; doi:10.1128/mSphere.00836-20)

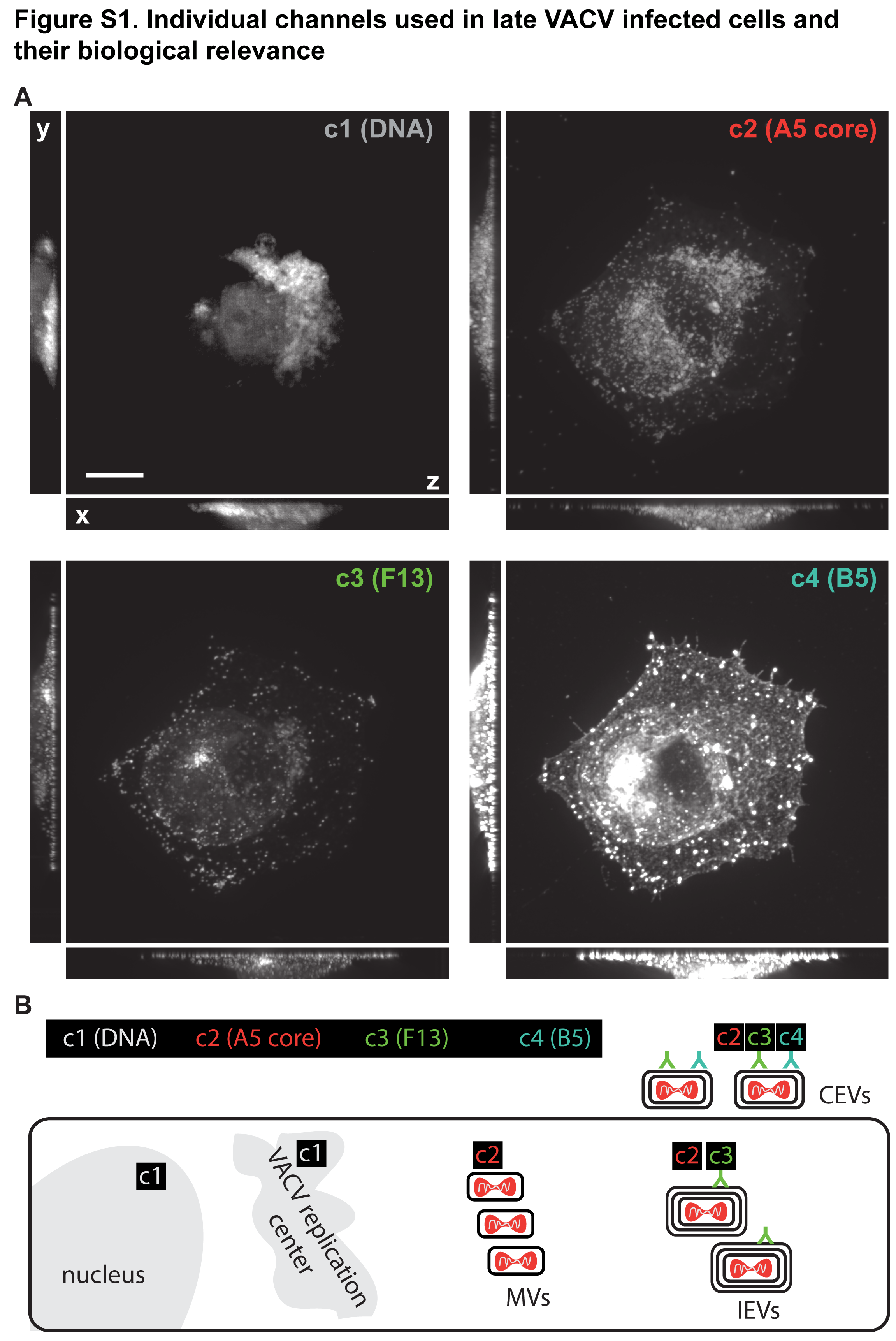

Supplement: FIG S1 [file mSphere.00836-20-sf001.tif]

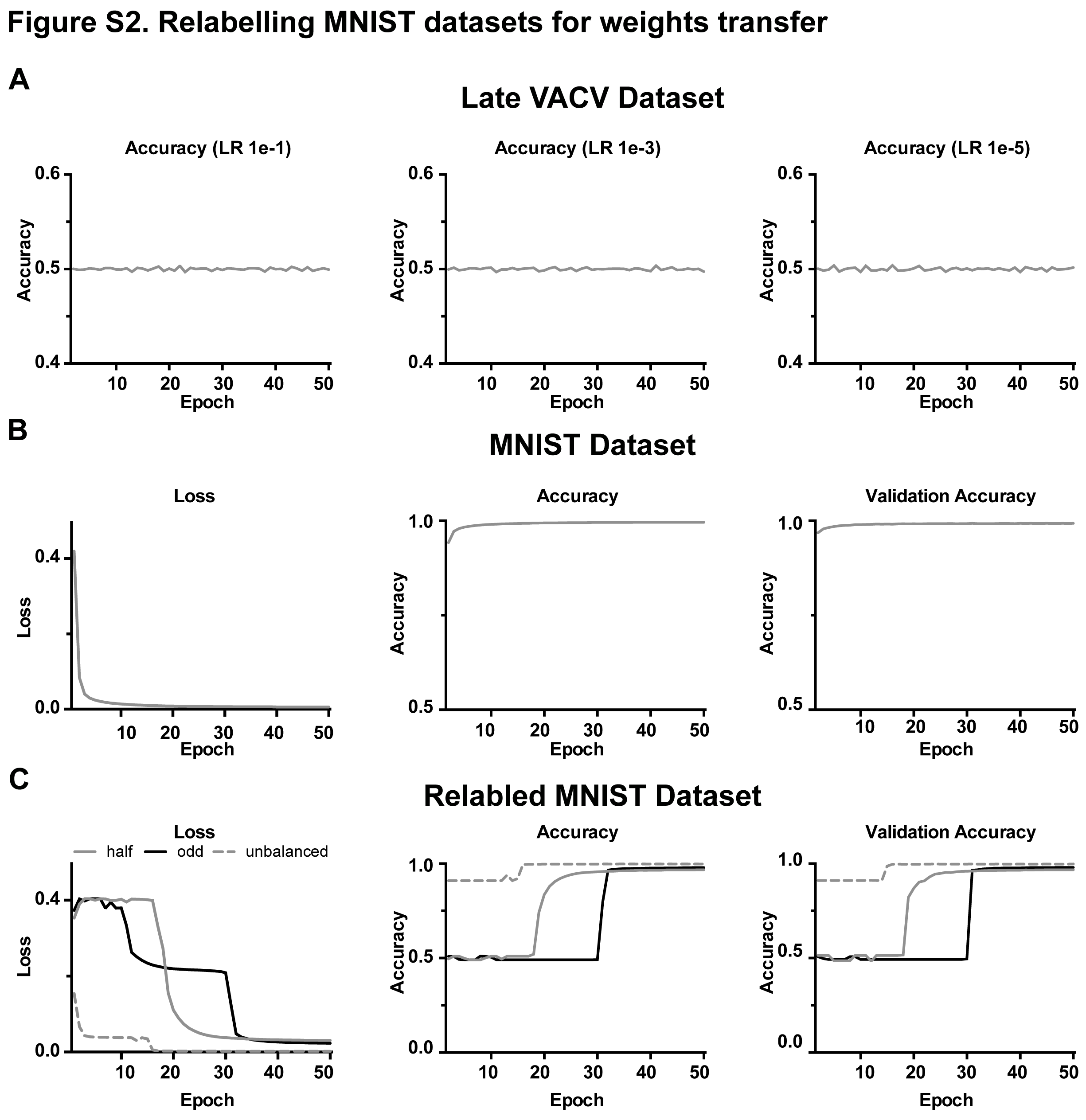

Supplement: FIG S2 [file mSphere.00836-20-sf002.tif]

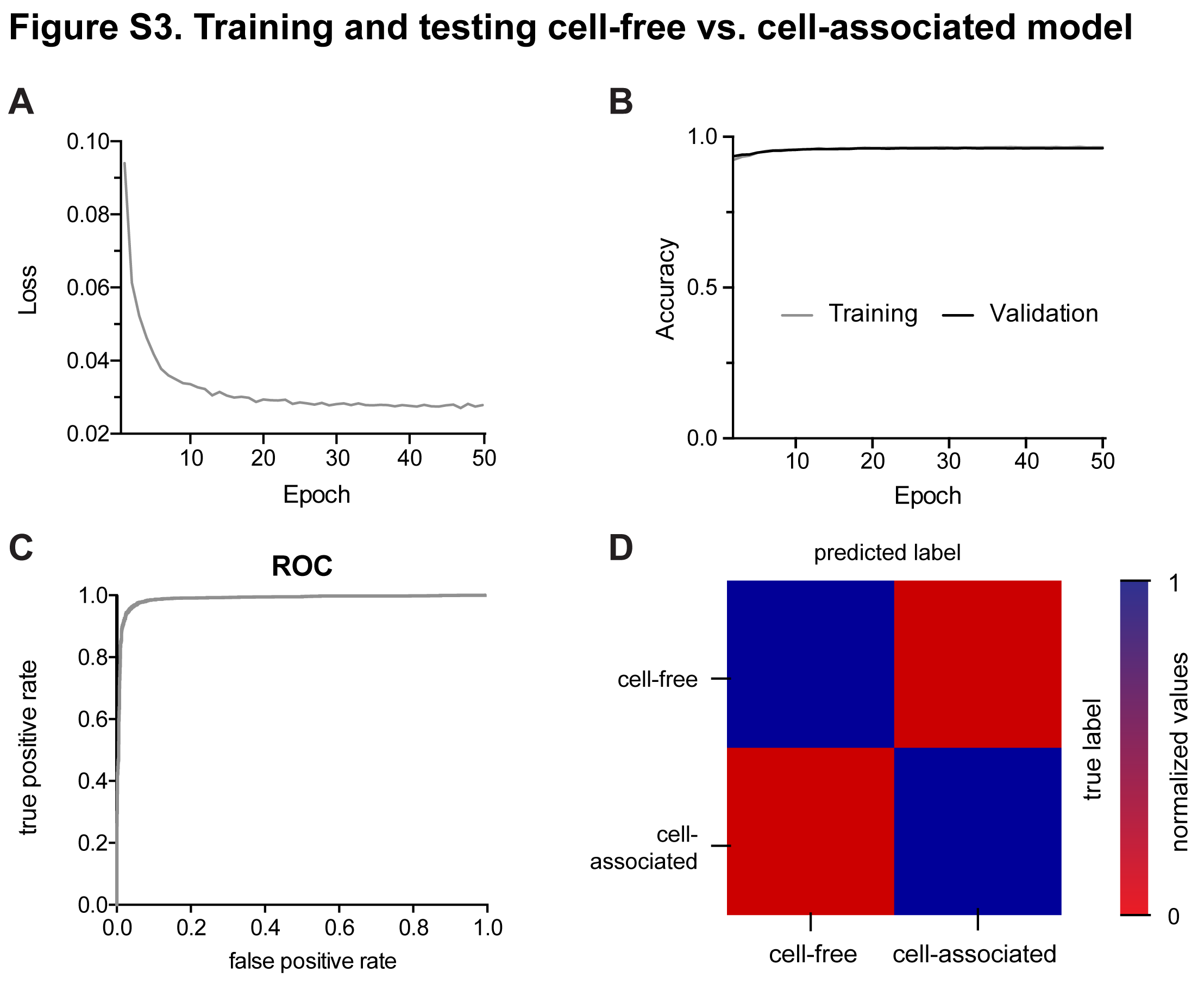

Supplement: FIG S3 [file mSphere.00836-20-sf003.tif]

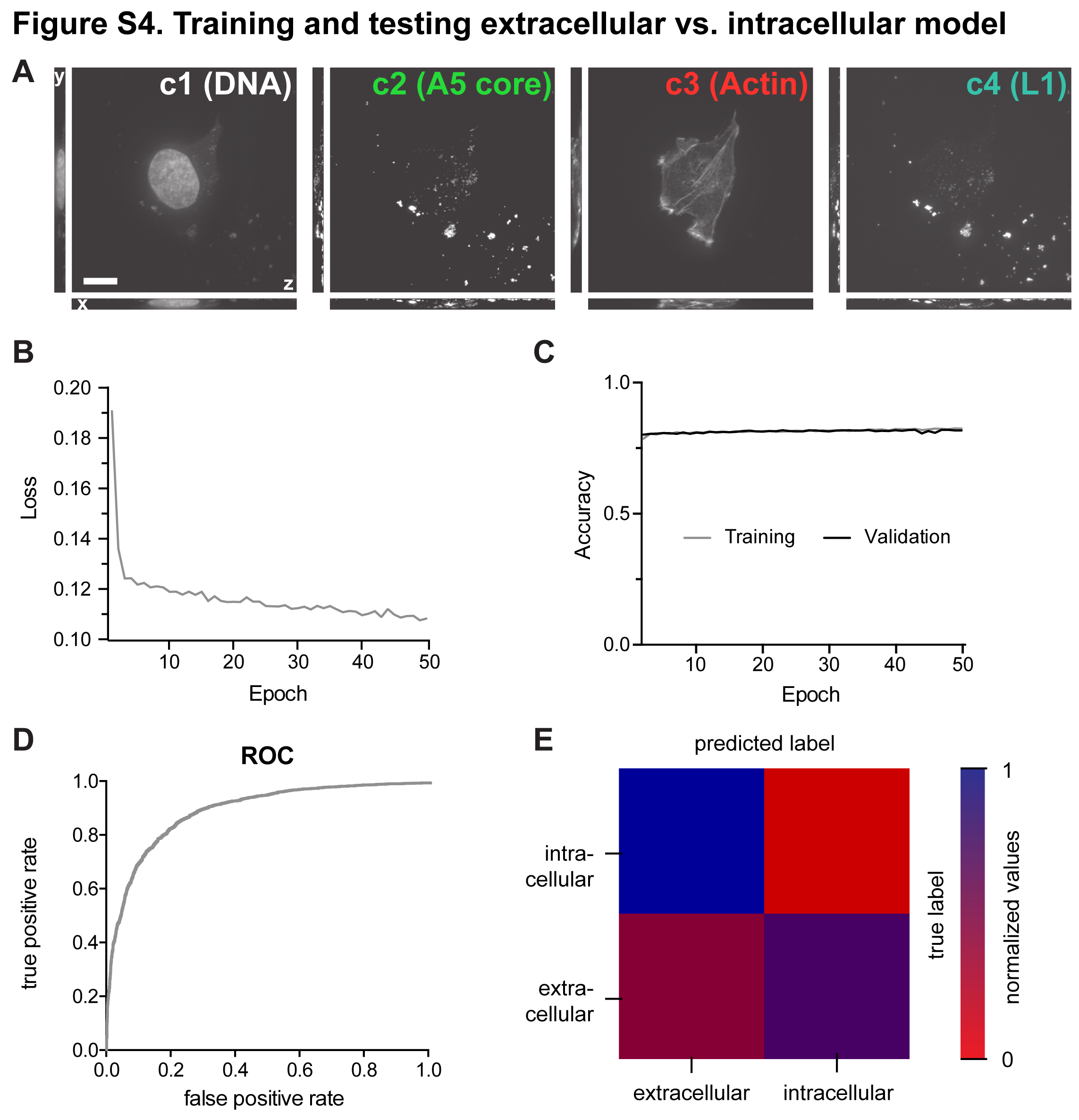

Supplement: FIG S4 [file mSphere.00836-20-sf004.tif]

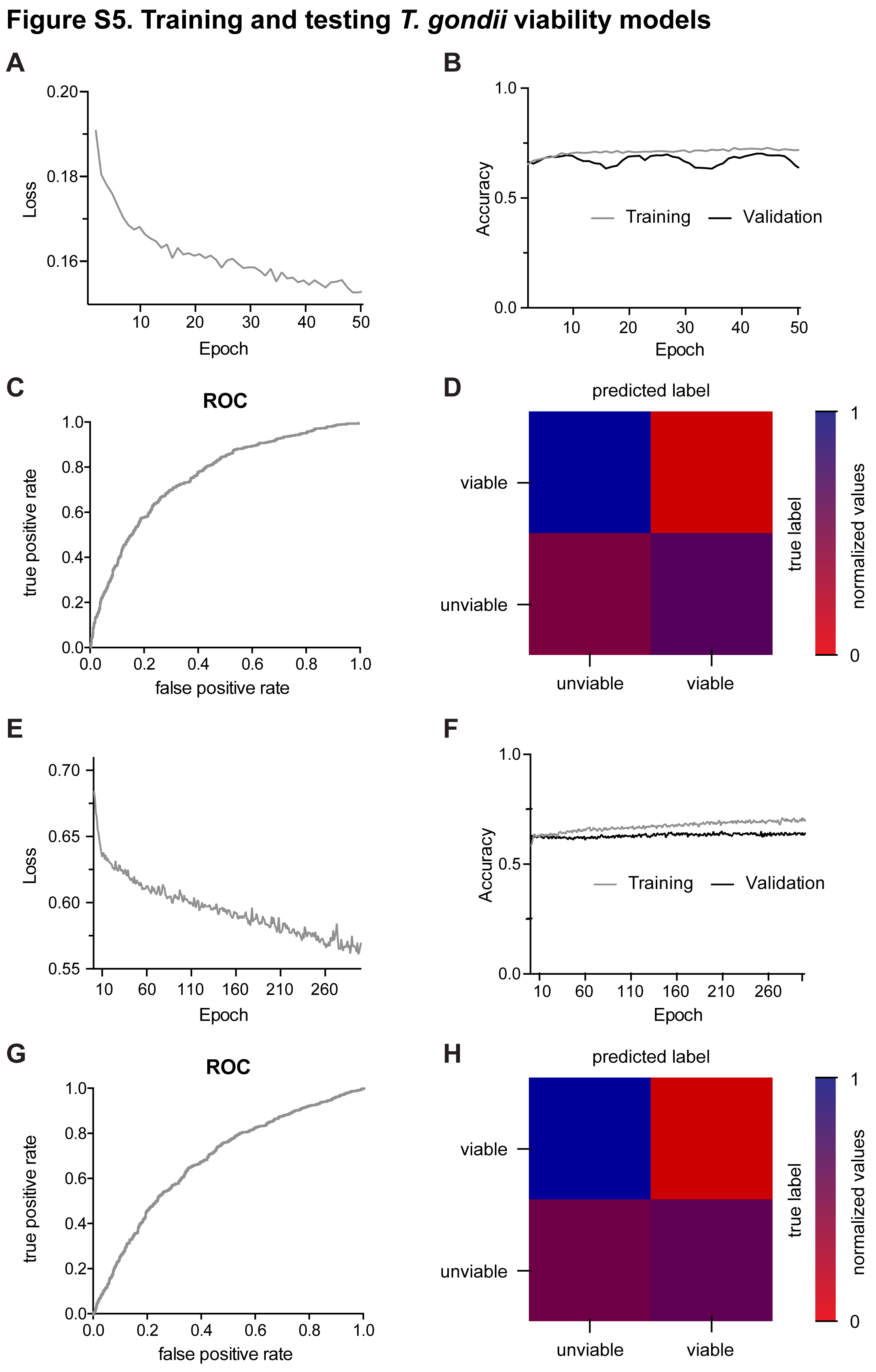

Supplement: FIG S5 [file mSphere.00836-20-sf005.tif]
